# Supplementary material for: A Genetically Encoded Fluorescent Biosensor for Intracellular Measurement of Malonyl-CoA
Source: ACS Bio Med Chem Au. 2024 Dec 18;5(1):184–93. doi: 10.1021/acsbiomedchemau.4c00103 (PMC11843332; doi:10.1021/acsbiomedchemau.4c00103)
Supplement: Supplementary file 1 — bg4c00103_si_001.pdf [file bg4c00103_si_001.pdf]

Supplemental Information

A Genetically Encoded Fluorescent Biosensor for Intracellular Measurement of Malonyl-CoA

Brodie L. Ranzau[1, 5], Tiffany D. Robinson[1, 5], Jack M. Scully[1], Edmund D. Kapelczak[2], Teagan S. Dean[1], Tara TeSlaa[2, 3] and Danielle L. Schmitt[1, 3, 4*]

[1]Department of Chemistry and Biochemistry, University of California, Los Angeles, Los Angeles, CA, 90095, USA

[2]Department of Molecular and Medical Pharmacology, David Geffen School of Medicine, University of California, Los Angeles, Los Angeles, CA, 90095, USA

[3]Molecular Biology Institute, University of California, Los Angeles, Los Angeles, CA, 90095, USA

[4]Institute for Quantitative and Computational Biosciences, University of California, Los Angeles, Los Angeles, CA, 90095, USA

[5]These authors contributed equally

*Corresponding author email: dlschmitt@chem.ucla.edu

Ranzau et al Supplemental Figure 1

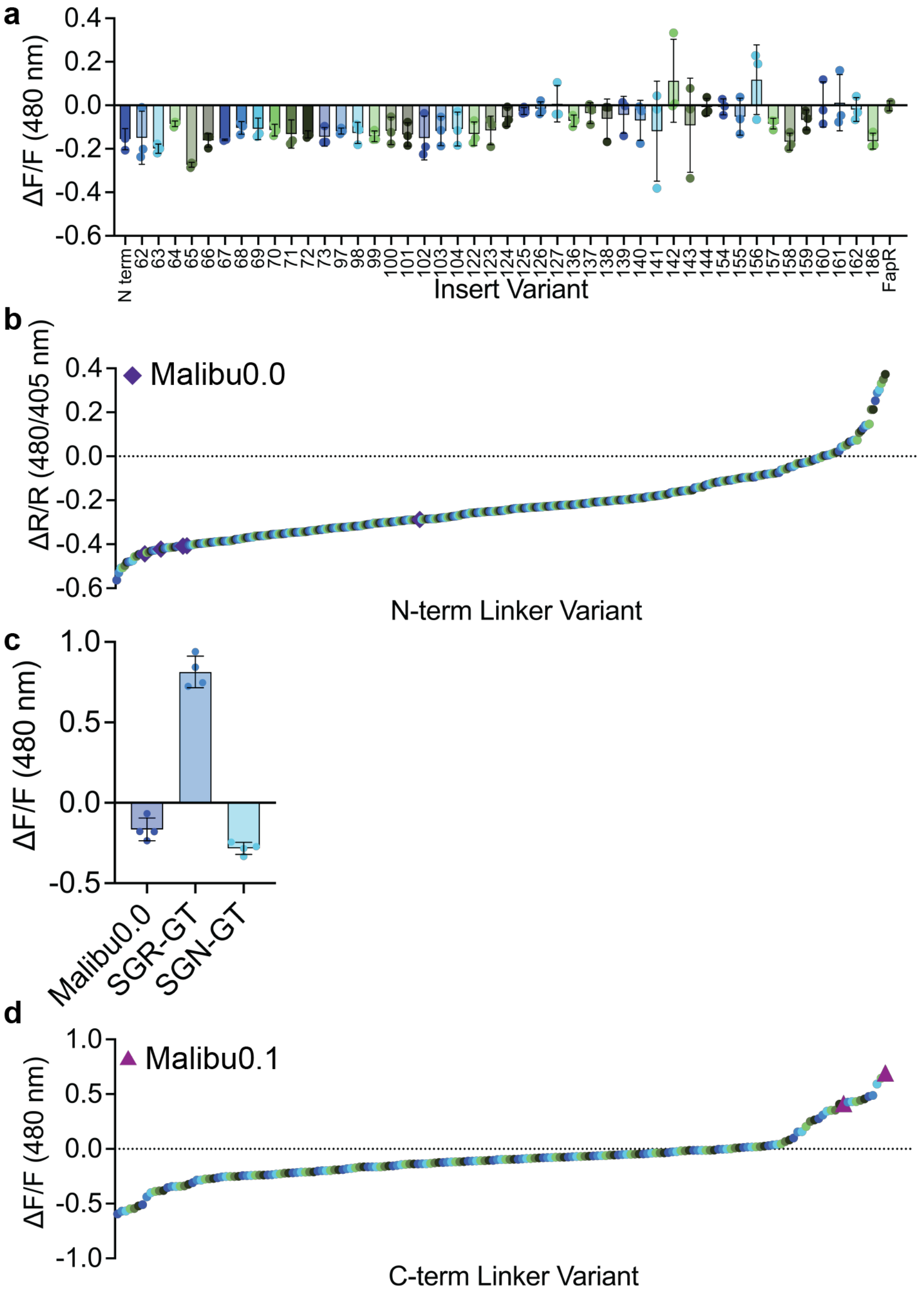

Supplemental Figure 1. Development of Malibu.

a, Fluorescence change (ΔF/F) after addition of 500 μM malonyl-CoA of initial Malibu variants in clarified bacterial lysate (n = 3 independent experiments).

b, Ratio change (ΔR/R) of N-terminal linker variants screened in clarified bacterial lysate, treated with 500 μM malonyl-CoA. Performance of Malibu0.0 (purple diamond) highlighted.

c, Fluorescence change (ΔF/F) of top N-terminal linker variants considered. Malibu0.0 has SAG-GT linkers (n = 2 independent experiments with 2 technical replicates).

d, Fluorescence change (ΔF/F) of C-terminal linker variants screened in clarified bacterial lysate, treated with 450 μM malonyl-CoA. Performance of Malibu0.1 (pink triangle) highlighted.

For all figures, dot plots show the mean ± SD.

Ranzau et al Supplemental Figure 2

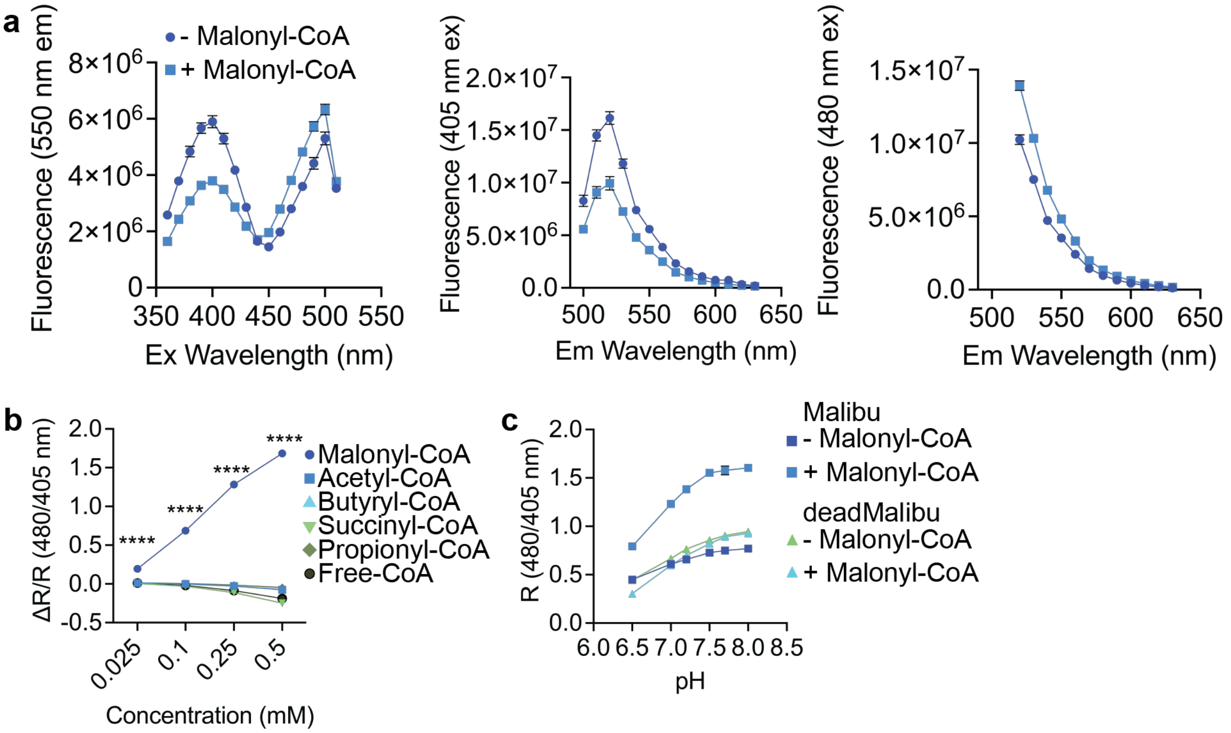

Supplemental Figure 2. Characterization of Malibu.

a, (left panel) Malibu excitation sweep from 360-510 nm excitation with fluorescence measured at 550 nm emission in the presence of either vehicle (dark blue) or malonyl-CoA (500 μM, light blue). (middle panel) Malibu emission sweep from 500-630 nm with excitation at 405 nm in the presence of either vehicle (dark blue) or malonyl-CoA (500 μM, light blue). (right panel) Malibu emission sweep from 520-630 nm with excitation at 480 nm in the presence of either vehicle (dark blue) or malonyl-CoA (500 μM, light blue). Data represents 1 protein preparation with 4 technical replicates.

b, Selectivity of Malibu towards malonyl-CoA, as measured by ratio change in bacterial lysate. Malibu was incubated with 0.025-0.5 mM of each respective CoA-containing molecule indicated. Data represent 2 independent experiments with 3 technical replicates each (****$p < 0.0001$, two way ANOVA with Tukey's multiple comparisons test).

c, Unnormalized pH dependency of either Malibu (squares) or deadMalibu (triangles) ratio changes in response to either vehicle (dark blue) or 500 μM malonyl-CoA (light blue) between pH 6.5-8. For Malibu, data are mean of 2 independent protein preparations with 4 technical replicates each. For deadMalibu, data are mean of 1 independent protein preparation with 3 technical replicates.

For all figures, plots show the mean ± SD.

Ranzau et al Supplemental Figure 3

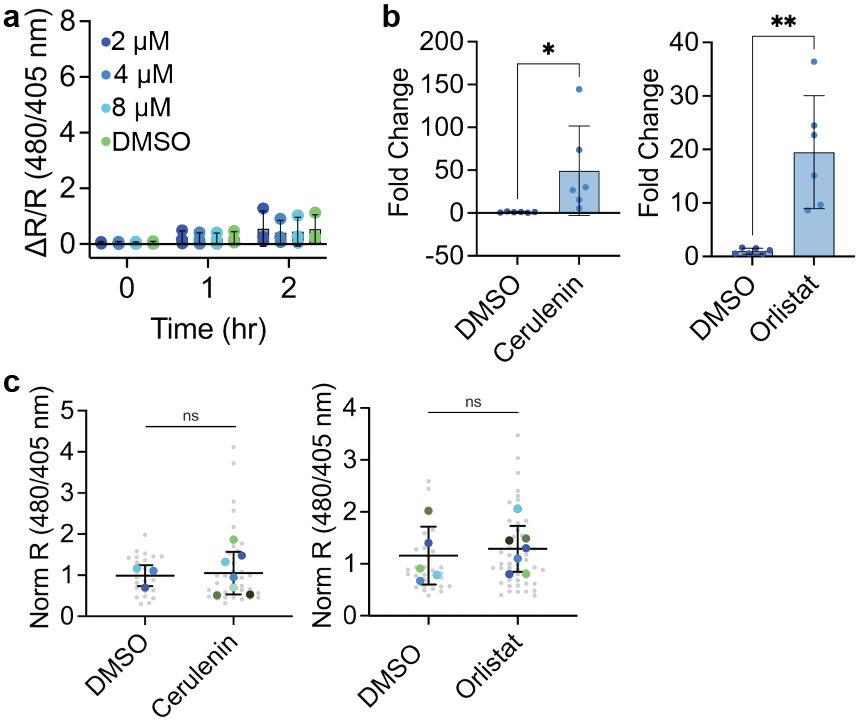

Supplemental Figure 3. Malibu reports malonyl-CoA dynamics in cells.

a, Ratio change of deadMalibu expressed in BL21 *E. coli* treated with either 2 μM cerulenin (dark blue, n = 3 independent experiments with 3 technical replicates each), 4 μM cerulenin (medium blue, n = 3 independent experiments with 3 technical replicates each), 8 μM cerulenin (light blue, n = 3 independent experiments with 3 technical replicates each), or DMSO (green, n = 3 independent experiments with 3 technical replicates each; 2-way ANOVA with Dunnett's multiple comparisons test, only statistical significance was indicated).

b, (left) Fold change of malonyl-CoA in HeLa cells after treatment with either DMSO (dark blue, n = 2 independent experiments with 3 technical replicates) or cerulenin (50 μM for 4 hr, light blue, n = 2 independent experiments with 3 technical replicates; *p = 0.0465, unpaired t-test). (right) Fold change of malonyl-CoA in HeLa cells after treatment with either DMSO (dark blue, n = 2 independent experiments with 3 technical replicates) or orlistat (15 μM for 12 hr, light blue, n = 2 independent experiments with 3 technical replicates; **p = 0.0016, unpaired t-test).

c, (left) Ratio change of deadMalibu fused to mScarlet (deadMalibu-mScarlet) expressed in HeLa cells treated with either DMSO (dark blue, n = 3 independent experiments) or cerulenin for 4 hr (50 μM, light blue, n = 7 independent experiments) normalized to mScarlet expression marker (ns, p =0.86, unpaired t-test). (right) Ratio change of deadMalibu-mScarlet expressed in HeLa cells treated with either DMSO (dark blue, n = 5 independent experiments) or orlistat for 12 hr (15 μM, light blue, n = 7 independent experiments) normalized to mScarlet expression marker (ns, p = 0.66, unpaired t-test).

For all figures, dot plots show the mean ± SD. For HeLa cell data, all cells are shown in small gray circles, each independent experiment average are shown in colored large circles, and the overall mean ± SD is plotted.

Ranzau et al Supplemental Table 1

Initial Designs	
Primer Number	Sequence 5' to 3'
NT_fwd_bb	GGCACAAGCTGGAGTACAACGGAACGGAGCTTTCCATACCTGAACTGAGAGAAAGAATTAAGAACGTGGC
NT_rev_bb	GCCTTGATATAGACGTTTCCCGCTGACATGGATCCCCATCGATCCTTATCGTCATCGTCGTACAGATCC
62_fwd_bb	GGCACAAGCTGGAGTACAACGGAACGGAGGACGAAGTGAAGTCCCTGTCACTTGATGAAGTTATCG
62_rev_bb	GCCTTGATATAGACGTTTCCCGCTGAAAGTGTTTTCTCTGCCACGTTCTTAATTCTTTCTCTCAGTTCAG
63_fwd_bb	GGCACAAGCTGGAGTACAACGGAACGGACGAAGTGAAGTCCCTGTCACTTGATGAAGTTATCG
63_rev_bb	GCCTTGATATAGACGTTTCCCGCTGACTCAAGTGTTTTCTCTGCCACGTTCTTAATTCTTTCTCTCAG
64_fwd_bb	GGCACAAGCTGGAGTACAACGGAACGGAAGTGAAGTCCCTGTCACTTGATGAAGTTATCGGAG
64_rev_bb	GCCTTGATATAGACGTTTCCCGCTGAGTCCTCAAGTGTTTTCTCTGCCACGTTCTTAATTCTTTCTCTC
65_fwd_bb	CACAAGCTGGAGTACAACGGAACGGTGAAGTCCCTGTCACTTGATGAAGTTATCGGAGAAATTATTGACC
65_rev_bb	GCCTTGATATAGACGTTTCCCGCTGATTCGTCCTCAAGTGTTTTCTCTGCCACGTTCTTAATTCTTTCTC
66_fwd_bb	GGCACAAGCTGGAGTACAACGGAACGAAGTCCCTGTCACTTGATGAAGTTATCGGAGAAATTATTGACC
66_rev_bb	GCCTTGATATAGACGTTTCCCGCTGACACTTCGTCCTCAAGTGTTTTCTCTGCCACG
67_fwd_bb	TCCCTGTCACTTGATGAAGTTATCGGAGAAATTATTGACCTTG
67_rev_bb	CTTCACTTCGTCCTCAAGTGTTTTCTCTGCCACG
67_fwd_insert	AAAACACTTGAGGACGAAGTGAAGTCAGCGGGAAACGTCTATATCAAGGCCGACAAGCAG
67_rev_insert	CCGATAACTTCATCAAGTGACAGGGACGTTCCGTTGTACTCCAGCTTGTGCCCCAGGATG
68_fwd_bb	GGCACAAGCTGGAGTACAACGGAACGCTGTCACTTGATGAAGTTATCGGAGAAATTATTGACCTTGAGC
68_rev_bb	GCCTTGATATAGACGTTTCCCGCTGAGGACTTCACTTCGTCCTCAAGTGTTTTCTCTGCCACG
69_fwd_bb	GGCACAAGCTGGAGTACAACGGAACGTCACTTGATGAAGTTATCGGAGAAATTATTGACCTTGAGCTGG
69_rev_bb	GCCTTGATATAGACGTTTCCCGCTGACAGGGACTTCACTTCGTCCTCAAGTGTTTTCTCTGCCACG
70_fwd_bb	GGCACAAGCTGGAGTACAACGGAACGCTTGATGAAGTTATCGGAGAAATTATTGACCTTGAGCTGGATG
70_rev_bb	GCCTTGATATAGACGTTTCCCGCTGATGACAGGGACTTCACTTCGTCCTCAAGTGTTTTCTCTGC
71_fwd_bb	GCACAAGCTGGAGTACAACGGAACGGATGAAGTTATCGGAGAAATTATTGACCTTGAGCTGGATGATCAG
71_rev_bb	GCCTTGATATAGACGTTTCCCGCTGAAAGTGACAGGGACTTCACTTCGTCCTCAAGTGTTTTCTCTGC
72_fwd_bb	GGCACAAGCTGGAGTACAACGGAACGGAAGTTATCGGAGAAATTATTGACCTTGAGCTGGATGATCAGGC
72_rev_bb	GCCTTGATATAGACGTTTCCCGCTGAATCAAGTGACAGGGACTTCACTTCGTCCTCAAGTGTTTTCTCTG

73_fwd_bb	GGCACAAGCTGGAGTACAACGGAACGGTTATCGGAGAAATTATTGACCTTGAGCTGGATGATCAGGC
73_rev_bb	GCCTTGATATAGACGTTTCCCGCTGATTCATCAAGTGACAGGGACTTCACTTCGTCCTCAAGTG
97_fwd_bb	GGCACAAGCTGGAGTACAACGGAACGGTGTTCAGCCGGAATCAGATTGCGAGAGGACACC
97_rev_bb	GCCTTGATATAGACGTTTCCCGCTGAGTGCTCCTGTTTTATTTCTAAAATGGATATCGCCTGATCATCC
98_fwd_bb	GGCACAAGCTGGAGTACAACGGAACGTTCAGCCGGAATCAGATTGCGAGAGGACACC
98_rev_bb	GCCTTGATATAGACGTTTCCCGCTGACACGTGCTCCTGTTTTATTTCTAAAATGGATATCGCCTGATC
99_fwd_bb	AGCCGGAATCAGATTGCGAGAGGACACCAT
99_rev_bb	GAACACGTGCTCCTGTTTTATTTCTAAAATGGATATCGCCTG
99_fwd_insert	GAAATAAAACAGGAGCACGTGTTCTCAGCGGGAAACGTCTATATCAAGGCCGACAAGCAG
99_rev_insert	TGTCCTCTCGCAATCTGATTCCGGCTCGTTCCGTTGTACTCCAGCTTGTGCCCCAGGATG
100_fwd_bb	GGCACAAGCTGGAGTACAACGGAACGCGGAATCAGATTGCGAGAGGACACCATTTATTTGCAC
100_rev_bb	GCCTTGATATAGACGTTTCCCGCTGAGCTGAACACGTGCTCCTGTTTTATTTCTAAAATGGATATCGCC
101_fwd_bb	GGCACAAGCTGGAGTACAACGGAACGAATCAGATTGCGAGAGGACACCATTTATTTGCACAGGC
101_rev_bb	GCCTTGATATAGACGTTTCCCGCTGACCGGCTGAACACGTGCTCCTGTTTTATTTCTAAAATGGATATCG
102_fwd_bb	GGCACAAGCTGGAGTACAACGGAACGCAGATTGCGAGAGGACACCATTTATTTGCACAGGCG
102_rev_bb	GCCTTGATATAGACGTTTCCCGCTGAATTCCGGCTGAACACGTGCTCCTGTTTTATTTCTAAAATGG
103_fwd_bb	GGCACAAGCTGGAGTACAACGGAACGATTGCGAGAGGACACCATTTATTTGCACAGGCGAAC
103_rev_bb	GCCTTGATATAGACGTTTCCCGCTGACTGATTCCGGCTGAACACGTGCTCCTGTTTTATTTCTAAAATGG
104_fwd_bb	GGCACAAGCTGGAGTACAACGGAACGGCGAGAGGACACCATTTATTTGCACAGGCGAACTCTTTGGC
104_rev_bb	GCCTTGATATAGACGTTTCCCGCTGAAATCTGATTCCGGCTGAACACGTGCTCCTG
122_fwd_bb	GGCACAAGCTGGAGTACAACGGAACGGATGACGAGCTGGCGCTGACTGCAAGTGC
122_rev_bb	GCCTTGATATAGACGTTTCCCGCTGAAATGACTGCAACGGCCAAAGAGTTCGCCTGTGC
123_fwd_bb	GGCACAAGCTGGAGTACAACGGAACGGACGAGCTGGCGCTGACTGCAAGTGCAGAC
123_rev_bb	GCCTTGATATAGACGTTTCCCGCTGAATCAATGACTGCAACGGCCAAAGAGTTCGCCTGTGC
124_fwd_bb	GAGCTGGCGCTGACTGCAAGTGCAGACATC
124_rev_bb	GTCATCAATGACTGCAACGGCCAAAGAGTTCG
124_fwd_insert	TTGGCCGTTGCAGTCATTGATGACTCAGCGGGAAACGTCTATATCAAGGCCGACAAGCAG
124_rev_insert	GATGTCTGCACTTGCAGTCAGCGCCAGCTCCGTTCCGTTGTACTCCAGCTTGTGCCCCAG
125_fwd_bb	GGCACAAGCTGGAGTACAACGGAACGCTGGCGCTGACTGCAAGTGCAGACATCCGC
125_rev_bb	GCCTTGATATAGACGTTTCCCGCTGACTCGTCATCAATGACTGCAACGGCCAAAGAGTTCG

126_fwd_bb	GGCACAAGCTGGAGTACAACGGAACGGCGCTGACTGCAAGTGCAGACATCCGC
126_rev_bb	GCCTTGATATAGACGTTTCCCGCTGACAGCTCGTCATCAATGACTGCAACGGCCAAAGAGTTCG
127_fwd_bb	GGCACAAGCTGGAGTACAACGGAACGCTGACTGCAAGTGCAGACATCCGCTTTACAAGACAGGTAAAGC
127_rev_bb	GCCTTGATATAGACGTTTCCCGCTGACGCCAGCTCGTCATCAATGACTGCAACGGC
136_fwd_bb	GGCACAAGCTGGAGTACAACGGAACGACAAGACAGGTAAAGCAGGGTGAACGTGTCGTAGCAAAAGCG
136_rev_bb	GCCTTGATATAGACGTTTCCCGCTGAAAAGCGGATGTCTGCACTTGCAGTCAGCG
137_fwd_bb	GGCACAAGCTGGAGTACAACGGAACGAGACAGGTAAAGCAGGGTGAACGTGTCGTAGCAAAAGCG
137_rev_bb	GCCTTGATATAGACGTTTCCCGCTGATGTAAAGCGGATGTCTGCACTTGCAGTCAGCGC
138_fwd_bb	GGCACAAGCTGGAGTACAACGGAACGCAGGTAAAGCAGGGTGAACGTGTCGTAGCAAAAGCG
138_rev_bb	GCCTTGATATAGACGTTTCCCGCTGATCTTGTAAAGCGGATGTCTGCACTTGCAGTCAGCGC
139_fwd_bb	GGCACAAGCTGGAGTACAACGGAACGGTAAAGCAGGGTGAACGTGTCGTAGCAAAAGCGAAAGTGACG
139_rev_bb	GCCTTGATATAGACGTTTCCCGCTGACTGTCTTGTAAAGCGGATGTCTGCACTTGCAGTCAGC
140_fwd_bb	GGCACAAGCTGGAGTACAACGGAACGAAGCAGGGTGAACGTGTCGTAGCAAAAGCGAAAGTGACGGC
140_rev_bb	GCCTTGATATAGACGTTTCCCGCTGATACCTGTCTTGTAAAGCGGATGTCTGCACTTGC
141_fwd_bb	GGCACAAGCTGGAGTACAACGGAACGCAGGGTGAACGTGTCGTAGCAAAAGCGAAAGTGACGGC
141_rev_bb	GCCTTGATATAGACGTTTCCCGCTGACTTTACCTGTCTTGTAAAGCGGATGTCTGCACTTGCAGTC
142_fwd_bb	GGCACAAGCTGGAGTACAACGGAACGGGTGAACGTGTCGTAGCAAAAGCGAAAGTGACGGC
142_rev_bb	GCCTTGATATAGACGTTTCCCGCTGACTGCTTTACCTGTCTTGTAAAGCGGATGTCTGCACTTGC
143_fwd_bb	GGCACAAGCTGGAGTACAACGGAACGGAACGTGTCGTAGCAAAAGCGAAAGTGACGGC
143_rev_bb	GCCTTGATATAGACGTTTCCCGCTGAACCCTGCTTTACCTGTCTTGTAAAGCGGATGTCTGC
144_fwd_bb	GGCACAAGCTGGAGTACAACGGAACGCGTGTCGTAGCAAAAGCGAAAGTGACGGC
144_rev_bb	GCCTTGATATAGACGTTTCCCGCTGATTCACCCTGCTTTACCTGTCTTGTAAAGCGGATGTCTGC
154_fwd_bb	GGCACAAGCTGGAGTACAACGGAACGGTCGAAAAAGAAAAAGGAAGAACGGTTGTCGAAGTGAACAGC
154_rev_bb	GCCTTGATATAGACGTTTCCCGCTGAAGCCGTCACTTTCGCTTTTGCTACGACACG
155_fwd_bb	GGCACAAGCTGGAGTACAACGGAACGGAAAAAGAAAAAGGAAGAACGGTTGTCGAAGTGAACAGC
155_rev_bb	GCCTTGATATAGACGTTTCCCGCTGAGACAGCCGTCACTTTCGCTTTTGCTACGACACGTTCACCCTGC
156_fwd_bb	GGCACAAGCTGGAGTACAACGGAACGAAAGAAAAAGGAAGAACGGTTGTCGAAGTGAACAGCTACG
156_rev_bb	GCCTTGATATAGACGTTTCCCGCTGATTCGACAGCCGTCACTTTCGCTTTTGCTACGACACG
157_fwd_bb	GGCACAAGCTGGAGTACAACGGAACGGAAAAAGGAAGAACGGTTGTCGAAGTGAACAGCTACG

157_rev_bb	GCCTTGATATAGACGTTTCCCGCTGATTTTCGACAGCCGTCACTTTCGCTTTTGCTACGACACG
158_fwd_bb	GGCACAAGCTGGAGTACAACGGAACGAAAGGAAGAACGGTTGTCGAAGTGAACAGCTACGTTGGC
158_rev_bb	GCCTTGATATAGACGTTTCCCGCTGATTCTTTTTCGACAGCCGTCACTTTCGCTTTTGCTACG
159_fwd_bb	GGCACAAGCTGGAGTACAACGGAACGGGAAGAACGGTTGTCGAAGTGAACAGCTACGTTGGCG
159_rev_bb	GCCTTGATATAGACGTTTCCCGCTGATTTTTCTTTTTCGACAGCCGTCACTTTCGCTTTTGCTACG
160_fwd_bb	GGCACAAGCTGGAGTACAACGGAACGAGAACGGTTGTCGAAGTGAACAGCTACGTTGGCG
160_rev_bb	GCCTTGATATAGACGTTTCCCGCTGATCCTTTTTCTTTTTCGACAGCCGTCACTTTCGCTTTTGC
161_fwd_bb	GGCACAAGCTGGAGTACAACGGAACGACGGTTGTCGAAGTGAACAGCTACGTTGGCG
161_rev_bb	GCCTTGATATAGACGTTTCCCGCTGATCTTCCTTTTTCTTTTTCGACAGCCGTCACTTTCGC
162_fwd_bb	GGCACAAGCTGGAGTACAACGGAACGGTTGTCGAAGTGAACAGCTACGTTGGCGAAG
162_rev_bb	GCCTTGATATAGACGTTTCCCGCTGACGTTCTTCCTTTTTCTTTTTCGACAGCCGTCACTTTCGC
186_fwd_bb	GGCACAAGCTGGAGTACAACGGAACGCATTCATAAGAATTCGAAGCTTGATCCGGCTGCTAACAAAGC
186_rev_bb	CTTGATATAGACGTTTCCCGCTGATTTTGAACGATACATGTCAAAGCGTCCAGAAAAAACAATTTCTTCG
fwd_insert	TCAGCGGGAAACGTCTATATCAAGGCCGACAAGC
rev_insert	CGTTCCGTTGTACTCCAGCTTGTGCCCCAGG
Further developments	
Primer Number	Sequence 5' to 3'
1	AATGCGTCTCTCGAATCANNKNNKAACGTCTATATCAAGGC
2	TAATCGTCTCATCACCGTTCCGTTGTACTC
3	AATGCGTCTCTGTGAAGTCCCTGTCACTTGATG
4	TAATCGTCTCATTCGTCCTCAAGTGTTTTCTC
5	AATGCGTCTCTGTGAAGTCCCTGTCACTTGATG
6	TAATCGTCTCATACCAGAACCCCGCATATGTATATCTC
7	AATGCGTCTCTGGTATGGCTAGCATGACTGG
8	TAATCGTCTCATCACMNNMNNGTTGTACTCCAGCTTGTGC
9	GAATTCGAAGCTTGATCCGGCTGCTAACAAAGCCCGAAAGGAAG
10	GGCCCACCGCGTTGCCGCTGCCGGTGCTCTGCAGTGAATGTTTTGAACGATACATG
11	GCAACGCGGTGGGCCAGGATACCCAGGAACGCGCCACCATGGTGAGCAAGGGCGAGGCAG
12	GCCGGATCAAGCTTCGAATTCTTACTTGTACAGCTCGTCCATGCCGCC
13	GAATTCTGCAGATATCCATCACACTGGCGGCCGCTCG
14	GATCCTTATCGTCATCGTCGTACAGATCCCGACCCATTTGC
15	GTACGACGATGACGATAAGGATCCCATGGAGCTTTCCATACCTGAACTGAGAGAAAGAAT
16	CACCGGCGGCATGGACGAGCTGTACAAGTAAGAATTCTGCAGATATCCATCACACTGGCG
17	GGCGGCATGGACGAGCTGTACAAGTAAGAATTCTGCAG

18	GTCCATGCCGCCGGTGGAGTGGCGGC
19	CAGATTGCGAGAGTACACCATTTATTTGCACAGGCGAACTC
20	GTGTACTCTCGCAATCTGATTCCGGCTGAACACGTG
21	CGTGGACTGCAAGTGCAGACATCCGCTTTACAAGACAGG
22	CACTTGCAGTCCACGCCAGCTCGTCATCAATGACTGC
23	GATTGCGGCAGGACACCATTTATTTGCACAGGCGAAC
24	GTCCTGCCGCAATCTGATTCCGGCTGAACACGTG
25	TACCGTCTCCCCCATGATGATGATGATGATGAGAACCCATATGTATATCTCCTTCTTAAAG TTAAACAAAATTATTTC
26	TACCGTCTCCTGGGGGCGGAGAGAATTTGTACTTTCAGGGAGGCGGAGGATCCATGGA GCTTTCCATACCTG
27	TACCGTCTCCCAGGGTGAACGTGTCGTAGC
28	TACCGTCTCCCCTGCTTTACCTGTCTTGTAAAGCG
